# Hydrogen-Terminated Diamond Surface as a Gas Sensor: A Comparative Study of Its Sensitivities

**DOI:** 10.3390/s21165390

**Published:** 2021-08-10

**Authors:** Michal Kočí, Alexander Kromka, Adam Bouřa, Ondrej Szabó, Miroslav Husák

**Affiliations:** 1 Department of Diamond and Associated Materials, Institute of Physics of the Czech Academy of Sciences, 162 00 Prague, Czech Republic; kromka@fzu.cz (A.K.); szabo@fzu.cz (O.S.); 2 Department of Microelectronics, Faculty of Electrical Engineering, Czech Technical University in Prague, 166 27 Prague, Czech Republic; bouraa@fel.cvut.cz (A.B.); husak@fel.cvut.cz (M.H.)

**Keywords:** nanocrystalline diamond (NCD), metal oxide (MO_X_), gas detectors

## Abstract

A nanocrystalline diamond (NCD) layer is used as an active (sensing) part of a conductivity gas sensor. The properties of the sensor with an NCD with H-termination (response and time characteristic of resistance change) are measured by the same equipment with a similar setup and compared with commercial sensors, a conductivity sensor with a metal oxide (MO_X_) active material (resistance change), and an infrared pyroelectric sensor (output voltage change) in this study. The deposited layer structure is characterized and analyzed by Scanning Electron Microscopy (SEM) and Raman spectroscopy. Electrical properties (resistance change for conductivity sensors and output voltage change for the IR pyroelectric sensor) are examined for two types of gases, oxidizing (NO_2_) and reducing (NH_3_). The parameters of the tested sensors are compared and critically evaluated. Subsequently, differences in the gas sensing principles of these conductivity sensors, namely H-terminated NCD and SnO_2_, are described.

## 1. Introduction

At present, great emphasis is placed on air quality and the detection of either toxic substances (NH_3_, CO, etc.) or substances that reduce the quality of life (CO_2_, etc.). Air quality is one of the significant properties to be monitored. The growing number of harmful substances released into the air, especially from industry, reduces the quality of life. Considering this, gas sensors, such as carbon monoxide, carbon dioxide, or nitrogen dioxide sensors, are almost essential for industry and everyday life. Over the last 20 years, the demand for quality and detection properties of sensors has increased significantly. For this reason, high demands are placed on the development of new types of sensors for the detection of various kinds of gases that achieve high accuracy, reproducibility, sensitivity, and stability. With the development of new materials and processing, smaller, more accurate, and cheaper sensors with a lower production cost can be developed. In order to increase a sensor’s performance, it is necessary to use novel sensing materials, surface modification, or new fabrication processes. Currently, great attention is paid to wide-bandgap (WBG) semiconductors. To date, the most used material for gas monitoring is MO_X_, especially SnO_2_ [1]. MO_X_ is used due to its low price and flexible production. On the other hand, these sensors have a very high operating temperature, between 300 and 450 °C [1,2].

Diamond is the most interesting material from the group of WBGs due to its ability to respond to oxidating and reducing gases, light illumination, temperature variation, and other surrounding conditions [3]. Diamond is a material that consists of carbon in the diamond crystal structure [4]. Due to a wide bandgap (5.5 eV), diamond was previously classified as an insulator. To prepare a quality semiconductor, it is necessary to modify the intrinsic diamond, for example, by doping it with foreign atoms. Natural diamonds have many foreign atoms (impurities) such as metals, nitrogen, etc. It is difficult to find the correct doping atoms because the diamond lattice constant is very small, only 3.57 Å (3.57 × 10^−10^ m) [3]. The most usual material is boron, which creates a p-type semiconductor [5,6]. There are several ways to prepare a synthetic diamond layer. The most used method is chemical vapor deposition (CVD) [6,7,8]. In most cases, hydrogen (>90%) and methane are present in a vacuum chamber, where they chemically react and form a thin layer of NCD on the substrate. The most used deposition technique is plasma-enhanced CVD (PECVD), or plasma-assisted CVD (PACVD), where plasma enhances the chemical reactions at lower temperatures. This method is not selective, and the layer grows on the whole substrate. The grown diamond is a hydrogen-terminated surface that reveals unique properties in induced subsurface p-type conductivity, also known as 2D hole gas (2DHG) [2,7,8,9]. Such a 2DHG top layer is sensitive to exposed gas or organic molecules [2,6]. The gas sensing properties of hydrogen-terminated diamond were explored thoroughly in previous works [2,6,7,9,10,11,12].

However, a comparison of a diamond-based gas sensor’s performance with other gas sensor types is still missing. The responses of the sensors with an NCD active layer prepared by PECVD, which can be a suitable alternative for commercial MO_X_ gas sensors, were measured in this work and compared with two commercial sensors (the conductivity (MO_X_) sensor TGS 826 and pyroelectric sensor PY2055). The conductivity sensor TGS 826 uses SnO_2_ as an active material, which is currently the most widely used detection material due to its low cost, sufficient responsivity, and easy adjustment for a given gas. The infrared pyroelectric sensor Pyreos PY2055 uses absorption spectrophotometry in the infrared region of the spectrum, which is very selective and slightly temperature-dependent. All these sensors were tested in oxidizing and reducing gas mixtures at concentrations up to 100 ppm.

## 2. Experimental

### 2.1. Experimental Setup for Gas Sensor Testing

**Figure 1:**
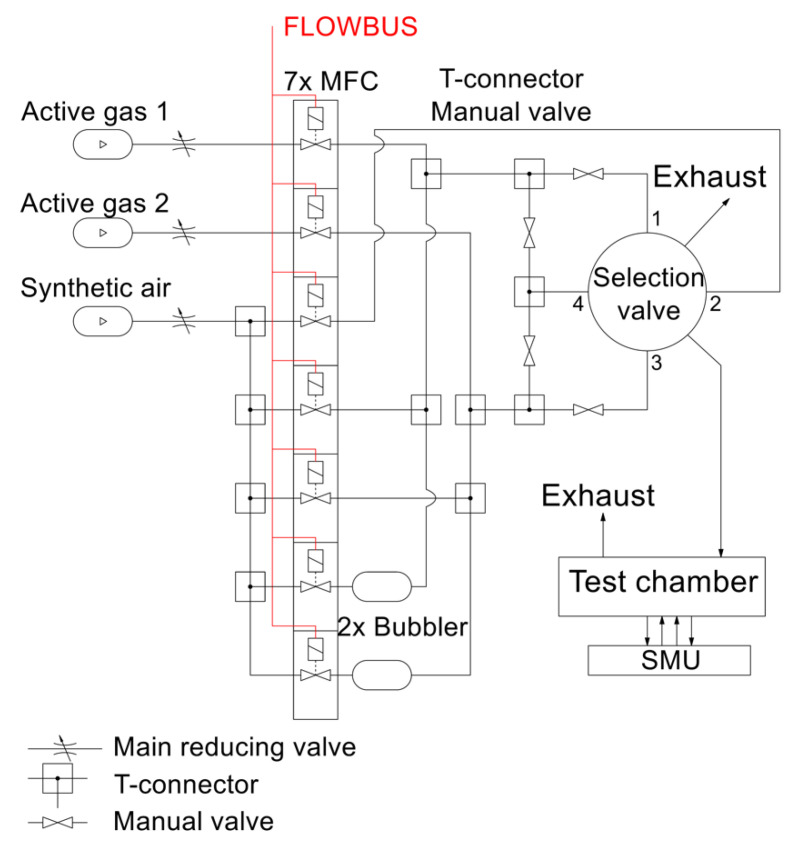
Schematic diagram of experimental setup for gas sensor testing.

**Figure 2:**
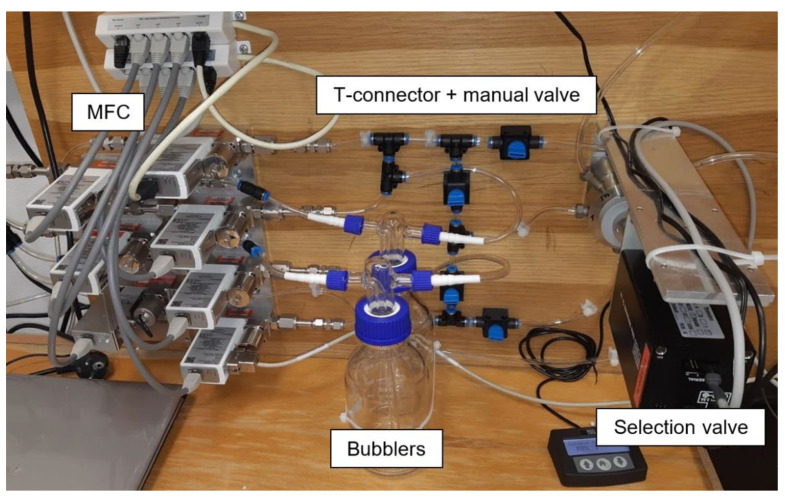
Photo of experimental setup for gas sensor testing.

**Figure 3:**
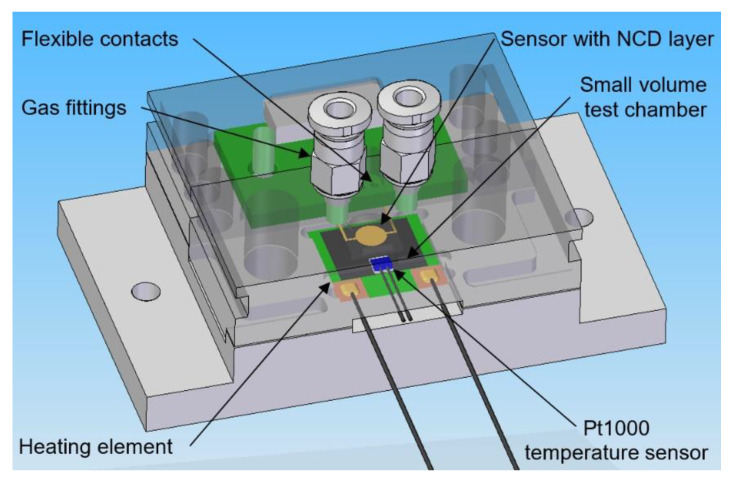
3D design of testing chamber for NCD sensors with H-termination.

The electrical parameters of the gas sensors are measured with a computer-controlled system consisting of mass flow controllers (MFCs), bubblers, a 4-input selection valve, a test chamber, a source measure unit (SMU), and data acquisition through a PC using the LabVIEW program, as shown in Figure 1 and Figure 2. Bronkhorst FG-201CV MFCs work on the principle of cooling the heating element using the gas flow. The FLOWBUS bus, which is based on the RS-485 bus, is used for communication with the control PC. At the same time, the bus provides electrical power to the flow meters. The MFCs are followed by a T-connector mixer system, which uses a turbulent mixing of gases and bubblers to control the humidity. The manual valves are used to reconfigure the system between two different mixtures and one mixture with two active gases. This system has the ability to supply two different mixtures via a Valco EUTA selection valve with 4 inputs and 2 outputs. For chamber flushing, synthetic air is used. An advantage of the system is its ability to create two different mixtures with different concentrations and humidity. The sensors with the H-terminated NCD layer are placed in the testing chamber (Figure 3). This chamber is designed for interdigital conductivity sensors. The testing chamber consists of a base, a rear part with flexible measuring contacts, a front part that allows the connection of a test mixture with a small test chamber with a volume of about 0.2 cm^3^ above the sensitive part of the sensor, and a temperature control assembly consisting of a heating element and a Pt1000 temperature sensor. Each part of the chamber is entered and attached by neodymium magnets and cut-outs in individual parts. This method allows quick and easy assembly and disassembly in case of a sensor replacement. This chamber and the Tektronix PWS4602 SMU allow two- or four-wire DC resistance measurements. In our case, the four-wire DC method was used. The advantage of using this SMU is the ability to choose the energy source, either current or voltage, and its value. A heating element is used to regulate the temperature. It heats the entire chamber and the sensor to a maximum temperature of 125 °C. A Pt1000 thermometer measures the current temperature.

**Figure 4:**
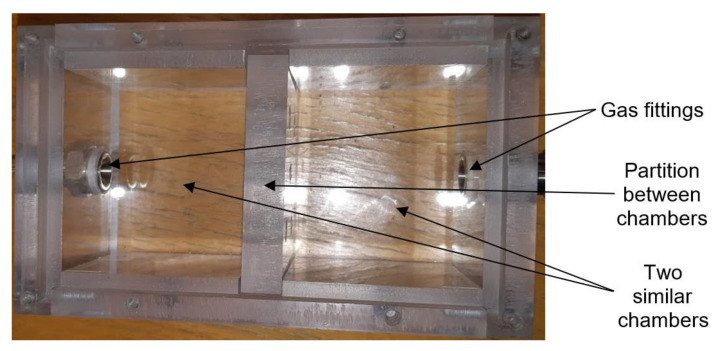
Photo of testing chamber for commercial sensors (top view).

The commercial sensors are placed in a universal test chamber made out of polycarbonate Macrolon (Figure 4) with two identical and separated sections and with the partition between the sections. The volume of one cell is 22 cm^3^. Its larger dimension aggravates the time characteristic, but it allows the installation and measurement of bigger commercial sensors, including the radiation source for the IR sensor PY2055.

### 2.2. Sensor Elements with H-Terminated NCD

**Figure 5:**
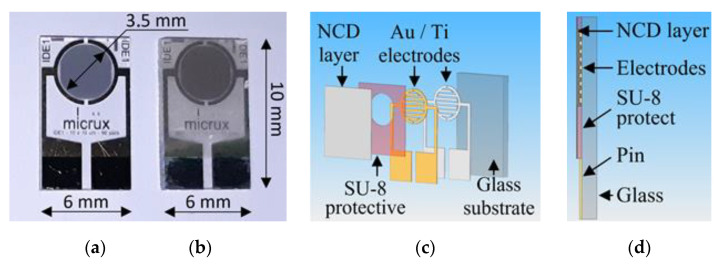
Photographs of (**a**) IDT structure on a glass substrate and (**b**) sensor with NCD active layer, (**c**) exploded schematic view, and (**d**) schematic cross-section.

The H-terminated NCD is grown using the microwave PECVD technique on the interdigital (IDT) structure ED-IDE1-Au with 90 pairs of 10/10/0.2 μm electrodes (10 μm width of the conductive gold-titanium electrodes, 10 μm gap between electrodes, and 200 nm thickness of electrodes) from Micrux Technologies. These IDT elements show good adhesion of gold electrodes to transparent glass substrates, which are thermally stable during the growth of the diamond layer at temperatures around 500 °C. Photographs and schematics of the diamond sensor are shown in Figure 5.

The preparation of the H-terminated NCD layer includes three parts: the preparation of the adhesive NCD layer, preparation of the final NCD layer, and functionalization of the layer by termination. In the first part of the deposition, an adhesive layer is optimized to prevent peeling off due to the different thermal expansion coefficients of diamond and the substrate. The deposition system, where the adhesive diamond interlayer is first prepared, is represented by two linear microwave antennas, which induce minimal thermal stress due to low temperatures (<400°C). This adhesive diamond layer was prepared at a thickness of 110 nm at low temperatures and a low deposition rate (about 4.4 nm/h). The process parameters of the system with linear antennas are the following: the power of the microwave (MW) generators = 1.7 kW, working pressure = 0.15 mbar of the gas mixture (150 sccm (standard cubic centimeters per minute) H_2_, 5 sccm CH_4_, 20 sccm CO_2_), deposition time = 25 h, and substrate temperature during deposition = 290 °C. In the second part, a final diamond layer is prepared in a focused plasma system. This system is characterized by a high diamond growth rate at higher temperatures (>500 °C). The diamond layer is prepared at a thickness of 150 nm (a deposition rate of about 50 nm/h). The process parameters of the focused plasma system are the following: the power of the MW generators = 1.5 kW, working pressure = 30 mbar of the gas mixture (300 sccm H_2_ and 3 sccm CH_4_), deposition time = 3 h, and maximal substrate temperature during deposition = 520 °C. After deposition, it is necessary to functionalize the diamond layer by termination. The NCD layers are plasma-activated in hydrogen microwave plasma immediately after the deposition in a focused plasma system for 20 min (the power of the MW generators = 1.5 kW, working pressure = 30 mbar at 500 °C). Both sensors were technologically processed at the same time.

**Figure 6:**
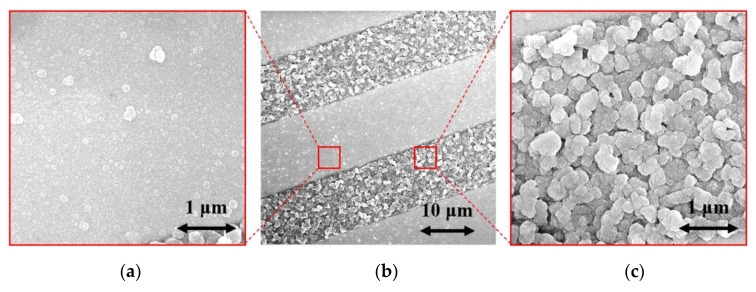
SEM images of (**a**) NCD on electrode, (**b**) NCD on IDT structure, and (**c**) NCD on glass.

**Figure 7:**
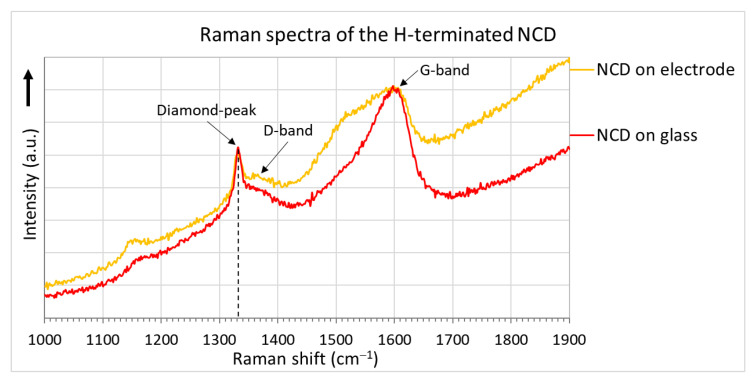
Raman spectra of the NCD layers.

The surface morphologies of the H-terminated NCD layer found using an SEM are shown in Figure 6. Figure 6b shows the surface of NCD on Au-Ti electrodes and glass (a gap between the electrodes). Figure 6a,c show enlarged surfaces of the sensor’s active layer. The figure on the left is the NCD layer above the electrode, and the NCD layer above the glass (a gap between the electrodes) is on the right. On both substrate material parts (glass and electrode), continuous thin films, revealing nanosized crystal features, are grown. Raman spectra from both of these parts (Figure 7) exhibit a narrow peak at 1332 cm^−1^ attributed to diamond, and two broad bands labelled D and G at 1350 and 1595 cm^−1^ are recognizable as disordered sp^2^ carbon phases and graphitic phases; a fingerprint of trans-polyacetylene segments located at the grain boundaries of the NCD films is visible at 1150 and 1500 cm^−1^ [13]. The quality of the hydrogen termination is indirectly verified by the measurement of the water contact angle. The H-terminated NCD is hydrophobic. A higher contact angle means more terminated hydrogen on the surface and thus a better response to the exposed gas. The minimal contact angle for good sensing properties is about 90° [8]. The fabricated layers revealed similar contact angles over 100° (104° for sensor No. 1 and 101° for sensor No. 2). However, the contact angle does not reflect the electronic quality of the induced p-type channel; therefore, both H-terminated NCD layers were tested, too.

### 2.3. Figaro TGS 826 Commercial Sensor

**Figure 8:**
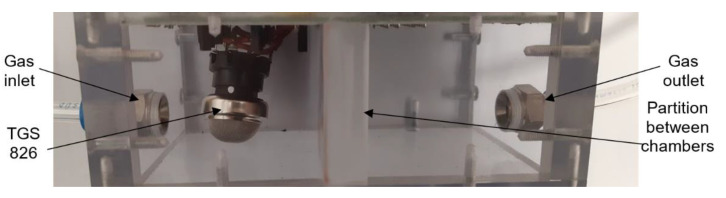
Photograph of conductivity sensor TGS 826 in test chamber.

The commercially available conductivity sensor TGS 826 from Figaro (Figure 8) is designed for the detection of ammonia (reducing gas) with a concentration from 30 ppm to 300 ppm [14]. A larger test chamber with approximately 110 times the volume of the previously described test chamber is used, because the TGS 826 is larger than the diamond sensor. This sensor uses SnO_2_ as an active material; it is currently the most widely used detection material due to its low cost, sufficient responsivity, and easy adjustment for a given gas [1,15]. According to the manufacturer’s datasheet, the conductivity of the active material increases with increasing ammonia concentration. This conductivity sensor monitors a change in the conductivity of the active layer due to chemisorption, i.e., the binding (sorption) of gas molecules on a solid’s surface by chemical bonding with electron transfer [15]. This sensor’s advantages are reproducibility and a manufacturer-defined response. It is the most common type of gas sensor for ammonia.

### 2.4. Pyreos PY2055 Commercial Sensor

**Figure 9:**
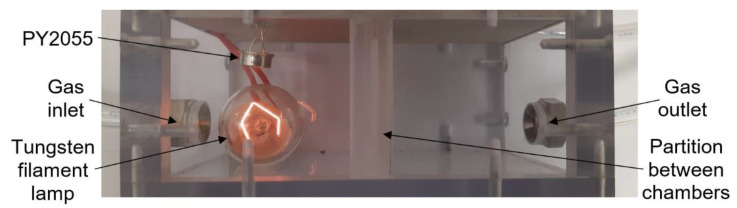
Photograph of infrared sensor PY2055 in test chamber.

The infrared pyroelectric sensor PY2055 from Pyreos (Figure 9) is used to detect nitrogen dioxide (oxidizing gas) in the mixture [16]. For this sensor type, it is necessary to use a source of mid-wavelength infrared light. In this test, a tungsten filament lamp is used to cover wavelengths from 3.9 to 6.2 µm. A pulse voltage at 10 Hz powers the bulb. For this reason, a large testing chamber is used for measurement. The sensor consists of two pyroelectric elements. A special optical filter is placed in front of each element; there is one filter for the gas absorption spectrum (6.2 μm) and one for the reference measurement (3.9 μm). Infrared gas sensors use absorption spectrophotometry in the infrared region of the spectrum. This detection method can determine the composition of a gas mixture or detect a specific type of gas [15]. The sensor’s output signals are represented by an alternating component of the output voltage modulated to half the supply voltage. The gas concentration is calculated as the AC component of Ch2 voltage divided by the AC component of Ch1 (REF) voltage [16]. The advantages of this sensor are the ability to take measurements without the active material coming into contact with the gas, accuracy, high selectivity using suitable filters, and a wide range of concentrations including up to almost 100% gas concentration.

## 3. Results

The responses of two H-terminated NCD conductivity sensors and two commercial sensors (TGS 826 and PY2055) are tested for sensitivity to ammonia and nitrogen dioxide gases.

### 3.1. H-Terminated NCD Conductivity Sensors

**Figure 10:**
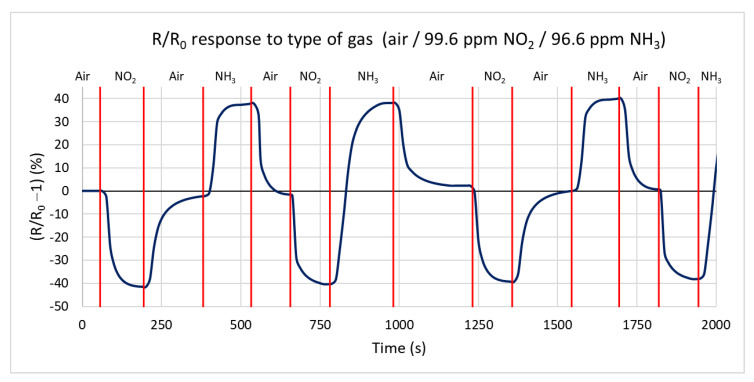
Time dependence of H-terminated NCD sensor’s response to three types of gases (ammonia, nitrogen dioxide, and synthetic air) at 125 °C with a gas flow rate of 100 sccm.

In the first measurement, the response of the H-terminated NCD conductivity sensor to two active gases, oxidizing and reducing, is measured in a test chamber at a temperature of 125 °C. Ammonia with a concentration of 96.6 ppm and nitrogen dioxide with a concentration of 99.6 ppm in a synthetic air mixture are used for testing. Figure 10 shows the percentual dependence of the steady-state resistance on time. Changing the gas to nitrogen dioxide, the resistance decreases by 41% from a steady value resistance R_0_ of 216 kΩ to 127 kΩ with a maximum speed of −0.998 kΩ/s. Changing the gas to ammonia caused an increase in the resistance of 39% from 216 kΩ to 302 kΩ, with a maximum speed of 0.908 kΩ/s. During the change of the gas from oxidizing to reducing, the resistance increases by 138%, from 127 kΩ to 303 kΩ. The maximum rate of change in resistance reaches 1.8 kΩ/s. The measurements show that the sample’s responses to both gases are almost identical, with opposite changes in the conductivity in accordance with the theoretical expectation. The influence of the chamber’s volume on the reaction time can be neglected because of its tiny volume and high gas flow of 100 sccm.

**Figure 11:**
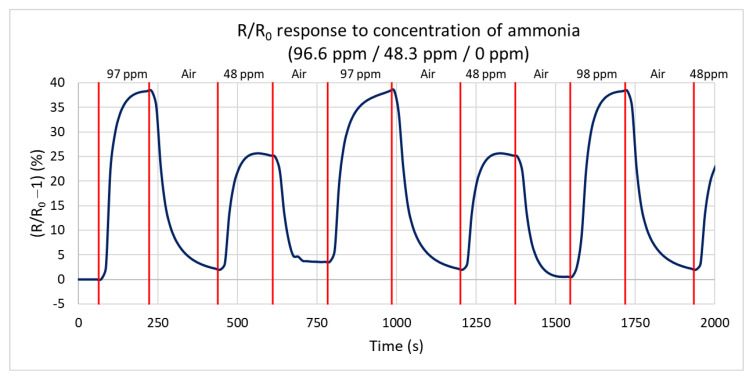
Time dependence of H-terminated NCD sensor’s response to three concentrations of ammonia (96.6 ppm, 48.3 ppm, and 0 ppm (synthetic air)) at 125 °C with a gas flow rate of 100 sccm.

Next, the response of the sensor to the reduction of the gas concentration at 125 °C is measured for ammonia concentrations of 96.6 ppm, 48.3 ppm, and 0 ppm (only synthetic air). Figure 11 shows the percentual dependence of the steady-state resistance on time. At a concentration of 96.6 ppm, the resistance increases by 38.5% from a steady value of 223 kΩ to 323 kΩ, in agreement with the first test. At a concentration of 48.3 ppm, the resistance decreases by 26% from a steady value of 224 kΩ to 294 kΩ. The difference of only 26% is caused by the non-linear sensitivity of NCD at a low concentration below 5 ppm. From these values, the calculated sensitivity is 0.26%/ppm, i.e., increasing the concentration by 1 ppm increases the resistance by 0.26%. The maximum rates of resistance change are very similar for repeated changes in the gas concentration (the sensor shows the repeatable dynamics of the response).

The response of the second sensor with an H-terminated NCD is measured with the same setup under the same conditions. The results are shown in Table 1. Subsequently, both sensors’ responses are measured at temperatures of 75 °C and 40 °C using the same setup.

### 3.2. TGS 826 SnO_2_ Conductivity Sensor

**Figure 12:**
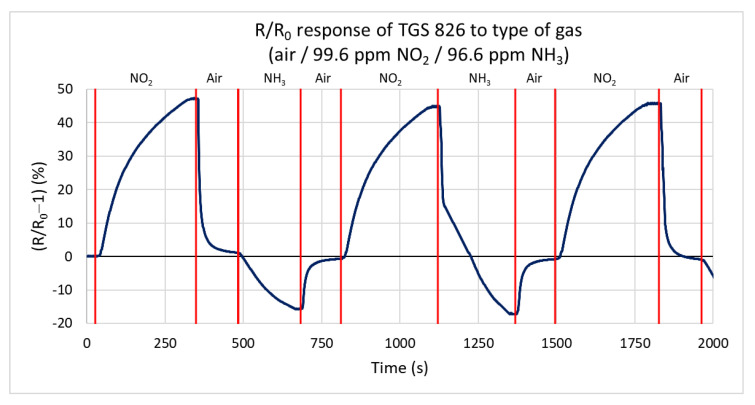
Response of TGS 826 sensor to three types of gases (ammonia, nitrogen dioxide, and synthetic air) at a gas flow rate of 100 sccm.

In this test, the response of the TGS 826 commercial sensor is measured for both gas types, oxidizing and reducing. This sensor is one of the most used commercial sensors for detecting and sensing ammonia. The sensor responds to both testing gases by changing the conductivity. Figure 12 shows the percentual dependence of the steady-state resistance on time. The action of the reducing gas decreases the resistance of the active layer, and the oxidizing gas increases the resistance. The sensor responds faster to ammonia (the primary gas that the sensor should detect) than to the oxidizing gas. The values for this test range from −16.9% to 47.8%. For nitrogen dioxide, the maximum measured rate of change of the resistance is 0.297 kΩ/s. The lower value is probably due to the higher volume of the test chamber. For ammonia, this value reaches −2.238 kΩ/s.

### 3.3. PY2055 Infrared Sensor

**Figure 13:**
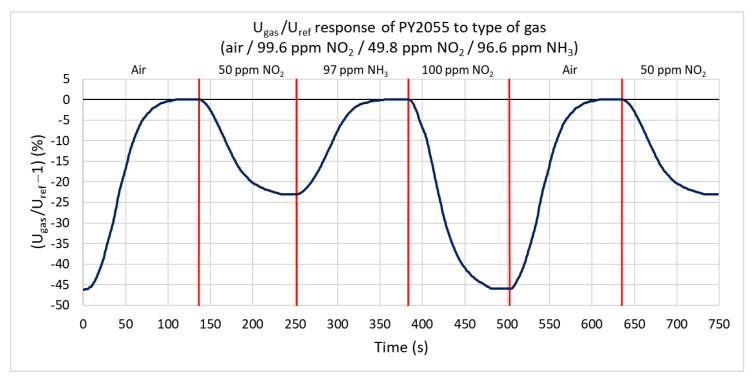
Response of PY2055 sensor to three types of gases (ammonia, nitrogen dioxide, and synthetic air) at a gas flow rate of 100 sccm.

Ammonia and nitrogen dioxide with maximum concentrations of 99.6 ppm are used to test the infrared sensor’s response. Figure 13 shows the percentual dependence of the output voltage’s root mean square (RMS) value on the reference signal. Nitrogen dioxide (oxidizing gas) absorbs the IR radiation and decreases the effective value of the output signal. The ratio does not change under the action of ammonia. The range of response values is from 0% to −46%. At 99.6 ppm nitrogen dioxide, the ratio decreases by 46%, and at a concentration of 49.8 ppm, it decreases by 23%. The sensitivity is −0.3146%/ppm. For nitrogen dioxide, the rate of change is only 1.7%/s. The lower value of this rate is mostly due to the higher volume of the test chamber.

### 3.4. Comparison of Sensors

The electronic properties and responses of the sensors are summarized in Table 1. The table includes the measured data for two commercial sensors and two conductivity interdigital sensors with H-terminated diamond active layers, which were heated to three different temperatures, 125, 75, and 40 °C. Bold values indicate the best value from each row.

The time necessary to reach the operating temperature of H-terminated diamond active layers is one minute, and the power consumption for the first measurement (the energy to heat up to operating temperature) is 125 W·s for 125 °C, 45 W·s for 75 °C, and only 20 W·s for 40 °C. Both sensor layers are created by the same technological process, but they exhibit different gas and temperature responses. These differences are most likely due to small differences in nucleation, deposition, and the structure of the thin layer (barriers between particles, etc.). The second sensor shows lower response to the gas at the temperature of 125 °C, but it can also be used at a low temperature of 40 °C, as shown in Table 1.

The first commercial sensor is the Figaro TGS 826, which uses SnO_2_ on the ceramic tube as an active material and a complementary principle similar to that of the diamond layer. The sensor consists of a 0.8 W heating element in the ceramic tube [14]. The total power consumption for one measurement is 240 W·s due to the 5 min of preheating necessary to reach the operating temperature of 300 °C [17]. The second sensor is the Pyreos PY2055: it uses absorption spectrophotometry in the infrared region of the spectrum, which is very selective. The total power consumption of this type of sensor depends on the IR radiation source used. A 1.2 W bulb with a 50% duty cycle was used in this case, and the total consumption is only 3 W·s.

## 4. Discussion

The designed and realized system is fully functional and suitable for testing sensors on two gas types, unlike most systems, which use only one active gas [6,9]. The selection valve allows fast switching between gases while keeping the flows constant, which allows a sufficiently continuous and defined measurement, minimizing peak-like events, in contrast to a system without a selection valve [6].

Experimental results show that the H-terminated NCD sensors are fully functional, with similar electrical characteristics to those of commercial sensors. However, the SnO_2_-based sensor has a faster response than the diamond-based sensors. The differences in sensor characteristics may be due to differences in the active material volume and the sensing material’s properties (i.e., surface morphology, etc.) [1,10]. The TGS 826 sensor consists of a ceramic tube with SnO_2_ on the surface, conductive electrodes, and a heating element ensuring the correct temperature, which is almost 200 °C higher (300 °C total) than the temperature of the H-terminated NCD sensors [17]. This arrangement allows gas access from all sides, unlike an interdigital structure with a thin layer. The other commercial sensor, PY2055, uses a different principle than the conductivity sensors. Infrared gas sensors use absorption spectrophotometry in the infrared region of the spectrum. This principle is suitable for gases formed by a more complex or asymmetric molecule; it shows a change in the molecule’s energy state [15]. IR sensors are not suitable for detecting gases with a monoatomic or symmetric diatomic molecule, as these gases do not absorb radiation in the infrared [15]. This sensor also needs an IR source and evaluation electronic components to compare it to conductivity sensors, which require only an ohmmeter or SMU. However, this sensor is highly selective, as shown in experimental measurements [15].

Both sensors with an H-terminated NCD layer were prepared using the same technological process. At such a low film thickness, the polycrystalline film is dominated by grain boundaries and defects localized at these boundaries. As a result, it is still complicated to create two almost identical diamond samples. We propose that the observed response differences should be assigned to inhomogeneities in the diamond crystals and traps localized at the diamond sub-surface. Using larger IDT dimensions, such differences should be suppressed but at lowered sensitivity. The fabrication reproducibility can be improved by better controlling the fabrication steps, such as by ensuring a more densely packed nucleation density, the exact cooling of layers during switching off, etc. Similarly, commercial production also faces some reproducibility issues; for example, the TGS 826 conductivity sensors are produced in series and are subsequently tested and sorted into eighteen groups [14] by nominal resistance and sensitivity to ammonia.

In addition to the exposed gas type, the sensor conductivities are sensitive to other physical and material parameters, which can further increase or reduce the sensitivity of the sensors to the gas. Among the physical quantities by which the impedance sensor characteristics are determined, light, humidity, and temperature are the most important factors [7,18]. The H-terminated diamond gas sensor’s temperature is crucial to its reaction dynamic because higher temperatures increase the reactivity with the gas and reduce the response to the intensity of light [18].

### 4.1. SnO_2_ Surface Gas Interaction Model

**Figure 14:**
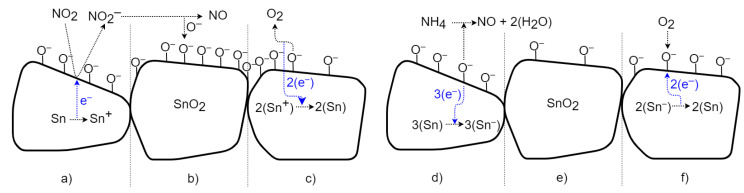
Schematic time process of gas interaction between the SnO_2_ material and oxidizing and reducing gases.

The change in conductivity in SnO_2_ is caused by chemisorption, reflecting the binding (sorption) of oxygen molecules on its solid surface by chemical bonding with electron transfer. Figure 14 depicts a schematic view of the time-sequenced set of interactions. The transfer of electrons between substances is called the oxidation-reduction process [1,15,19]. In the case of the oxidizing gas, NO_2_ (Figure 14 left), free electrons are removed; thus, the conductivity of the n-type semiconductor is reduced. In Figure 14a, NO_2_ gas molecules are adsorbed on the surface of the SnO_2_ bulk; the gas attacks the available Sn sites and removes electrons from the conduction band, forming NO_2_^−^. This increases the barrier between particles and reduces conductivity. Subsequently, in Figure 14b, molecules of NO_2_^−^ desorb as NO, leaving binding oxygen ions behind [19,20]. After exposure to a non-oxidizing gas (in Figure 14c), chemisorbed oxygen molecules with negative charges on the surface are released as O_2_ with a neutral charge, and electrons are returned to SnO_2_ [20]. This returns the conductivity to its previous value. Exposing the SnO_2_ surface to the reducing gas NH_3_ (Figure 14 right) transfers free electrons into the material and increases the conductivity of the n-type semiconductor. In Figure 14d, molecules of NH_4_ are adsorbed, react with binding oxygen ions, and create charge-neutral NO and H_2_O (air humidity) molecules. The excess electrons from the oxygen ions are transferred into semiconductors (in Figure 14e). This increases the conductivity and reduces the barrier between particles [20]. After exposure to a non-reducing (i.e., air) gas (in Figure 14f), the conductivity returns to the previous value due to the sorption of two oxygen ions from the neutral O_2_ and the removal of free electrons [20].

### 4.2. H-Terminated NCD Surface Gas Interaction Model

**Figure 15:**
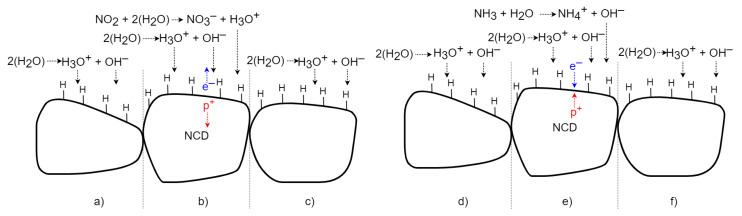
Schematic time process of gas interaction between the H-terminated NCD and oxidizing and reducing gases.

H-terminated NCD reveals a similar detection principle to the surface of SnO_2_ but with the opposite effect on its surface conductivity. The change in the NCD surface conductivity does not work on the gas sorption principle: it involves chemical reactions forming counter ions on its surface via an electron transfer model [7]. A widely established H-terminated diamond surface doping mechanism was used to explain the sensing mechanism. A thin layer of adsorbed water from the air is formed on the diamond surface [11]. The water molecule dissociates the ions H_3_O^+^ and OH^−^. The H_3_O^+^ ions attract electrons from the diamond surface, so the p-type surface conductivity is formed on the H-terminated NCD (Figure 15a,d)). If oxidizing gas molecules (NO_2_) are present (Figure 15b), the concentration of H_3_O^+^ ions rises due to a set of chemical reactions of the oxidizing gas with the adsorbed water monolayer. This causes a superiority of H_3_O^+^ molecules and creates a charge imbalance. Electrons are transferred from the diamond top surface to the direction of positive ions. Next, the resultant holes increase the 2DHG conductivity [2,10,11]. After exposure to a non-oxidizing (air) gas (Figure 15c), the number of H_3_O^+^ ions decreases and again H_3_O^+^ equilibrates with OH^−^. The electrons return to the diamond, and conductivity reduces to its original value [10]. In the case of a reducing gas, such as NH_3_ (Figure 15e), the concentration of OH^−^ ions increases due to the set of chemical reactions of the reducing gas with the adsorbed water monolayer. Due to the higher number of NH_4_^+^ ions, the concentration of the ions decreases, and electrons are shifted to the diamond and partially neutralize the 2DHG, which finally reduces the diamond’s surface conductivity [10,12]. After exposure to a non-reducing (air) gas (Figure 15f), the number of OH^−^ ions decreases and OH^−^ equilibrates with H_3_O^+^ again. The electrons return from the diamond, and the conductivity increases to the original value.

## 5. Conclusions

An NCD layer with H-termination was used as the active layer of the conductivity sensors. The crystallographic morphology of the prepared sensors with H-terminated NCD thin layers was confirmed by SEM and Raman spectroscopy. The SEM showed a continuous diamond layer on the electrode and glass, and the Raman spectra exhibit a sharp diamond peak for both parts. Fabricated H-terminated NCD sensors revealed sensor characteristics comparable to two commercial sensors in a similar testing setup after they were exposed to the reducing and oxidizing gases. The fabricated sensors have smaller dimensions and require a shorter amount of time for the first measurement, and thus they require less energy than the commercial TGS 826. Still, the TGS 826 sensor had a faster response to ammonia, due to a larger active surface area and a geometrical arrangement that allowed the gas access from all sides [17]. The infrared PY2055 sensor exhibits the highest selectivity, but it requires an IR source, which increases the consumption of electrical energy and demands on the size of the sensor system. It reacts only to nitrogen dioxide (selective gas), as declared in the manufacturer’s datasheet [16]; this was confirmed by measurements. The lower value of the time response is due to the higher volume of the test chamber. Overall, it is possible to conclude that hydrogen-terminated diamond expands the family of wide-bandgap semiconductors, where gas detection is possible even at temperatures of 100 °C. Moreover, its surface sensitivity can be enhanced not only by geometrical design (IDT distance) or surface morphology (nanorods) but also by using diamond-based composites, metal oxides, or transition metal dichalcogenide monolayers operated at low temperatures, with a reliable, reproducible response tuned to specific gas sensing applications.

## Figures and Tables

**Table 1:** Comparison of the responses and properties of the H-terminated NCD sensors with the TGS 826 and PY2055 commercial sensors.

	IDT Sensors with H-Terminated Diamond			Commercial Sensors	
	Temp. (°C)	Sensor No. 1	Sensor No. 2	Figaro TGS 826 (SnO_2_)	Pyreos PY2055 (IR Sensor)
R_0_ (kΩ)	125	216	111	52.9	0.12 (V)
	75	223	107		
	40	219	113		
(R-R_0_)/R_0_ response to 96.6 ppm NH_3_ (%)	125	39	13	−16.9	N.A. ((U-U_0_)/U_0_)
	75	4	5		
	40	N.A.	2.5		
(R-R_0_)/R_0_ response to 99.6 ppm NO_2_ (%)	125	−41	−11	47.8	−46 ((U-U_0_)/U_0_)
	75	−4.5	−7		
	40	N.A.	−5		
Time response to 96.6 ppm NH_3_ (Ω/s)	125	908	183	−2238	N.A.
	75	84	123		
	40	N.A.	83		
Time response to 99.6 ppm NO_2_ (Ω/s)	125	−998	−375	297	1.7 (%/s)
	75	−75	−214		
	40	N.A.	−102		
Sensitivity to NH_3_ (%/ppm)	125	0.259	0.092	−0.135	N.A.
Sensitivity to NO_2_ (%/ppm)	125	−0.161	−0.058	0.482	−0.315

## Data Availability

The data presented in this study are available on request from the corresponding author.
